# Spatio-temporal analysis of malaria incidence in the Peruvian Amazon Region between 2002 and 2013

**DOI:** 10.1038/srep40350

**Published:** 2017-01-16

**Authors:** Veronica Soto-Calle, Angel Rosas-Aguirre, Alejandro Llanos-Cuentas, Emmanuel Abatih, Redgi DeDeken, Hugo Rodriguez, Anna Rosanas-Urgell, Dionicia Gamboa, Umberto D´Alessandro, Annette Erhart, Niko Speybroeck

**Affiliations:** 1 Instituto de Medicina Tropical Alexander von Humboldt, Universidad Peruana Cayetano Heredia, Lima 31, Perú; 2 Research Institute of Health and Society (IRSS), Université catholique de Louvain, Brussels 1200, Belgium; 3 Department of Biomedical Sciences, Institute of Tropical Medicine, Antwerp 2000, Belgium; 4 Dirección Regional de Salud Loreto DIRESA Loreto, Loreto 160, Perú; 5 Departamento de Ciencias Celulares y Moleculares, Facultad de Ciencias y Filosofia, Universidad Peruana Cayetano Heredia, Lima 31, Perú; 6 Disease Control and Elimination, Medical Research Council Unit, Fajara 220, The Gambia; 7 London School of Hygiene & Tropical Medicine, London WC1E 7HT, UK; 8 Department of Public Health, Institute of Tropical Medicine, Antwerp 2000, Belgium

## Abstract

Malaria remains a major public health problem in the Peruvian Amazon where the persistence of high-risk transmission areas (*hotspots*) challenges the current malaria control strategies. This study aimed at identifying significant space-time clusters of malaria incidence in Loreto region 2002–2013 and to determine significant changes across years in relation to the control measures applied. Poisson regression and purely temporal, spatial, and space-time analyses were conducted. Three significantly different periods in terms of annual incidence rates (AIR) were identified, overlapping respectively with the pre-, during, and post- implementation control activities supported by PAMAFRO project. The most likely space-time clusters of malaria incidence for *P. vivax* and *P. falciparum* corresponded to the pre- and first two years of the PAMAFRO project and were situated in the northern districts of Loreto, while secondary clusters were identified in eastern and southern districts with the latest onset and the shortest duration of PAMAFRO interventions. Malaria in Loreto was highly heterogeneous at geographical level and over time. Importantly, the excellent achievements obtained during 5 years of intensified control efforts totally vanished in only 2 to 3 years after the end of the program, calling for sustained political and financial commitment for the success of malaria elimination as ultimate goal.

In 2014, the Peruvian Ministry of Health (MoH) recorded 65,239 malaria cases for the whole country, the majority of which were due to *Plasmodium vivax* (84%) and from the Amazon Region of Loreto (~90%)^1^. Malaria in this region has always been a major public health problem^2^ as shown in the literature back until the 1940 s. In 1944, reports indicated that approximately one third of the total malaria cases in the country occurred in the Amazon (about ~30,000 malaria cases of a total of 95,000). During the 50’s and 60’s, the eradication campaign implemented countrywide resulted in a drastic reduction of the total annual incidence to approximately 1,500 malaria cases in 1963^2^. Afterwards, malaria incidence remained at low levels until the early 90’s, before increasing steadily until 1997 when Loreto experienced the most important malaria epidemic with a total of 158,115 reported cases, 34% of which were caused by *P. falciparum*. The resurgence of malaria was associated with the reintroduction of *Anopheles darlingi* as malaria vector in the Amazon region^3^ and the presence of resistant *P. falciparum* strains to chloroquine (CQ) and sulfadoxine-pyrimethamine (SP), which in 2001 prompted the change in the national treatment policy to artemisinin-combination therapies (ACTs)^4^.

After the epidemic, the annual incidence dropped markedly and remained between 45,000 and 55,000 cases from 2000 to 2005. It was then followed by a steady decrease until 2010 and 2011 when only 11,504 and 11,793 cases were reported, respectively, following the scale-up of comprehensive control activities supported by the Global Fund Malaria Project “PAMAFRO”^5^. Since 2011, following the reduction of financial support allocated to malaria control activities, the number of malaria cases is alarmingly rising again in Loreto, with the total number of reported *P. falciparum* and *P. vivax* malaria cases increasing from 2,473 to 7,911 and from 9,306 to 35,826 cases, respectively, between 2011 and 2013^1^.

Many factors have been reported to significantly influence malaria transmission in Loreto^3^,^6^,^7^ and their relative contributions might have been different across districts and years in the past decade. Ecological characteristics facilitating breeding sites of *An. darlingi (i.e.,* rivers, fish ponds, climatic changes including El Niño phenomenon) or resting places for adult mosquitos (*i.e.,* surrounding vegetation, deforestation, housing characteristics) are thought to be the main factors for malaria transmission^6^,^7^,^8^,^9^. In addition, conditions that increase exposure to infectious mosquitos’ bites (*e.g.,* forest-based economical activities)^10^, and human behavioural factors that limit the coverage and effectiveness of malaria control interventions (*i.e.*, low use of preventive measures, inappropriate treatment seeking behaviours, and low treatment adherence) may also influence the malaria transmission^3^,^11^,^12^. Besides the above mentioned factors, the timing and the intensity of malaria control interventions may also play an important role in determining the geographical and chronological heterogeneity of malaria transmission.

Until now, available spatio-temporal analysis tools for the detection of malaria clusters^13^,^14^,^15^, have rarely been applied to analyse the Peruvian Amazon setting. This study aimed at analysing trends in malaria incidence in Loreto between 2002 and 2013, and identifying significant space-time district-clusters of malaria incidence in relation to applied control efforts.

## Results

### Trends in annual malaria incidence rates

**Figure 1:**
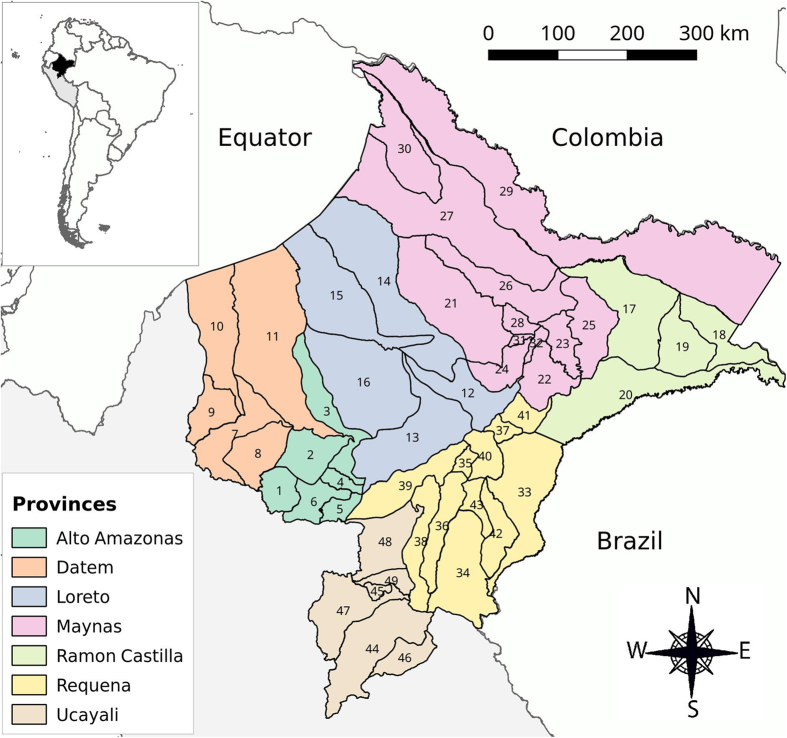
Map of Loreto Region with provinces and respective districts. Map generated with QGIS 2.16 (QGIS Development Team, 2016. QGIS Geographic Information System. Open Source Geospatial Foundation Project. http://www.qgis.org/) Districts’ names: 1: Balsapuerto, 2: Jeberos, 3: Lagunas, 4: Santa Cruz, 5: Teniente Cesar Lopez, 6: Yurimaguas, 7: Barranca, 8: Cahuapanas, 9: Manseriche, 10: Morona, 11: Pastaza, 12: Nauta, 13: Parinari, 14: Tigre, 15: Trompeteros, 16: Urarinas, 17: Pebas, 18: Ramon Castilla, 19: San Pablo, 20: Yavari, 21: Alto Nanay, 22: Fernando Lores, 23: Indiana, 24: SanJuan, 25: Las Amazonas, 26: Mazan, 27: Napo, 28: Punchana, 29: Putumayo, 30: Torres Causana, 31: Iquitos, 32: Belen, 33: Yaquerana, 34: AltoTapiche, 35: Capelo, 36: Emilio San Martin, 37: Jenaro Herrera, 38: Maquia, 39: Puinahua, 40:Requena, 41: Saquena, 42: Soplin, 43: Tapiche, 44: Contamana, 45: Inahuaya, 46: Padre Marquez,47: Pampa Hermosa, 48: Sarayacu, 49: Vargas Guerra.

**Figure 2:**
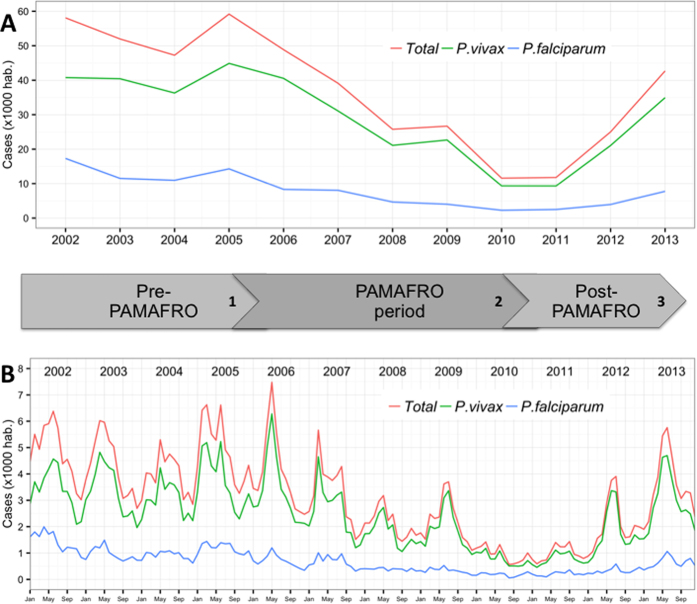
Annual (A) and monthly (B) malaria (overall and per species) incidence per 1000 population in Loreto Region: 2002–2013. Numbers in the timeline differentiate three periods: *1)* Pre-PAMAFRO period (Jan. 2002–Sept. 2005): control activities essentially based on passive case detection (PCD), and the response to malaria outbreaks with focalized active detection of symptomatic cases (ACD) and indoor residual spraying; *2)* PAMAFRO period (Oct. 2005–Dec. 2010): intensified comprehensive control strategies including the strengthening of malaria diagnosis and detection, improvement of malaria case-management, use of insecticide treated nets, and encouragement of community participation in environmental management; *3)* Post-PAMAFRO period (Jan. 2011–Dec. 2013): control activities essentially limited to passive case detection (PCD) at health facilities and irregular ACD in communities with high incidence of malaria cases.

Between 2002 and 2013, a total of 424,176 confirmed malaria cases were reported in Loreto Region (Fig. 1), including 334,713 (79%) *P. vivax* and 89,463 (21%) *P. falciparum* cases. The evolution of the overall- as well as species-specific- annual incidence rates (AIRs) are shown in Fig. 2A and Table 1. Annual malaria incidence rates steadily decreased by 80% between 2006 and 2010, from 48.9 to 11.6/1000, resulting in annual IRRs decreasing from 0.84 to 0.20 compared to 2002. Between 2011 and 2013, the AIR significantly increased from 11.8 to 42.7/1000 and the annual IRRs from 0.20 to 0.73. It is noteworthy that in the last 6 months of the PAMAFRO project, between July 2010 and February 2011, the monthly incidence rates (MIR) remained below 1.0/1000 (Fig. 2B). Both the *P. vivax* and *P. falciparum* AIRs followed a similar bi-phasic pattern from 2005 onwards (Table 1).

At district level, the AIRs were associated with the number of malaria control interventions implemented even after adjusting for environmental variables (*i.e.,* mean temperature, rainfall, humidity), population characteristics (*i.e.,* population density, population living in rural areas, population living in poverty) and number of health facilities in the district. Indeed, Loreto districts covered simultaneously with the two main strategies (*i.e.,* strengthening of malaria diagnosis/case management and LLINs distribution) (Adjusted IRR = 0.51, 95% CI: 0.45–0.57) and those covered with only one of them (Adj. IRR = 0.81, 95% CI: 0.73–0.89), at any given year, had significantly lower total AIRs compared to districts not covered with any intervention, and this was similar for *P. vivax* (Adj. IRR = 0.52, 95% CI: 0.47–0.59) and *P. falciparum* (Adj. IRR = 0.43, 95% CI: 0.37–0.51).

### Purely temporal cluster analysis

Results of the purely temporal cluster analysis at regional level and per year are shown in Table 2. Overall, significant temporal clusters of increased monthly malaria incidence (IRR range: 1.36–2.18) were identified every year for a 5 to 6-month period, usually from February to July, with occasional shifts of +/−1 month, except in the last three years when this cluster consistently started 2–3 months later and tended to last only for 3–4 months. This pattern was essentially the same for *P. vivax* during the 12-year period and for *P. falciparum* from 2002 to 2007. From 2008 onwards, the *P. falciparum* temporal clusters tended to be much shorter (≤4 months) with an extreme of 1-month cluster in July 2012. At district level, the temporal analysis identified even greater variations in cluster’s size and timing within and between districts (Supplementary Table S1).

### Purely spatial and time-space clusters

**Figure 3:**
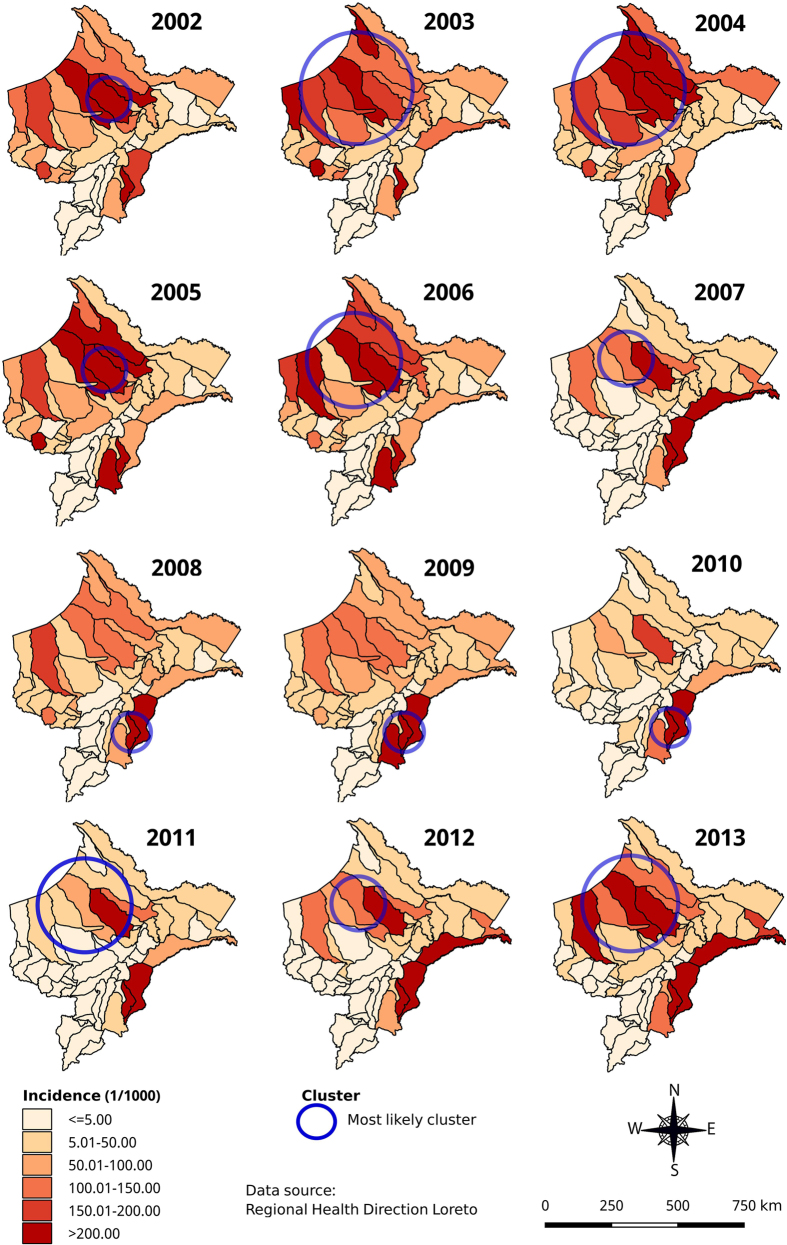
Annual overall (*P. vivax* and *P. falciparum*) malaria incidence and adjusted most significant spatial clusters at district level in Loreto Region: 2002–2013 (using Satscan purely spatial analysis). Map generated with QGIS 2.16 (QGIS Development Team, 2016. QGIS Geographic Information System. Open Source Geospatial Foundation Project. http://www.qgis.org/).

**Figure 4:**
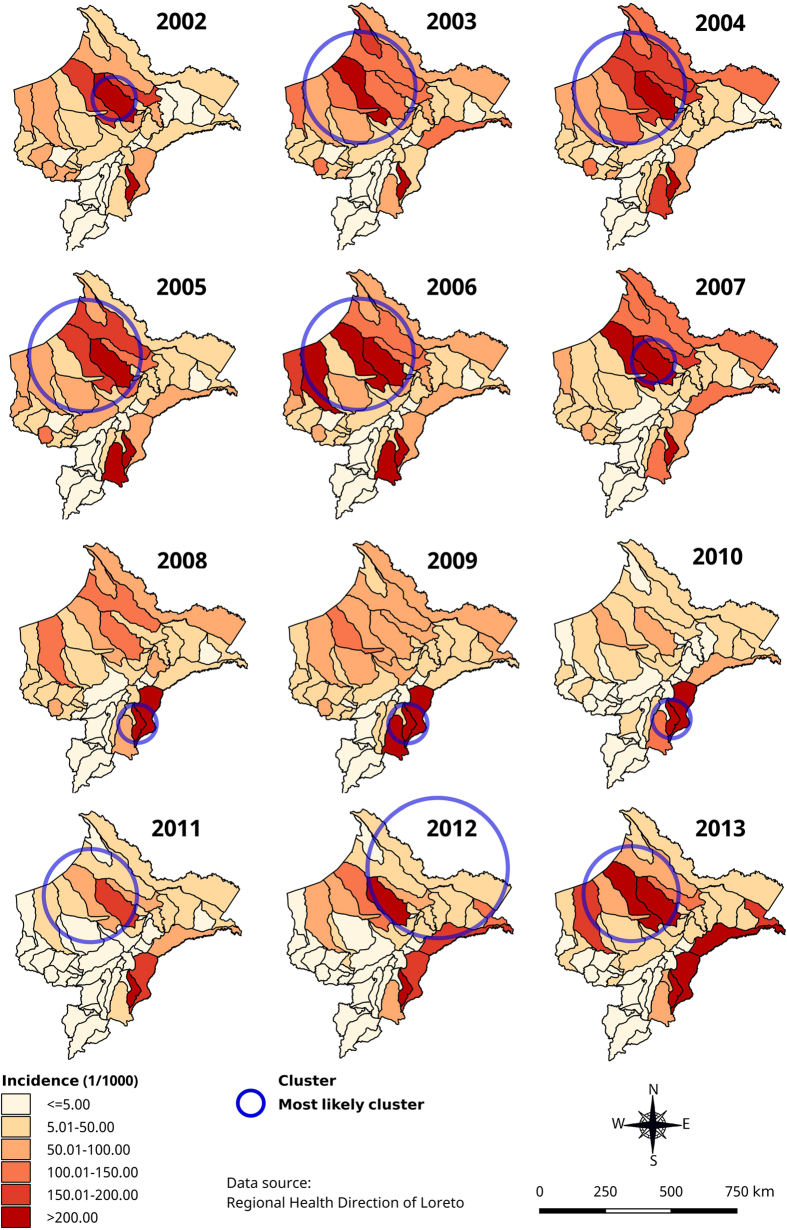
Annual *P. vivax* incidence and adjusted significant most likely spatial clusters at district level in Loreto Region: 2002–2013 (using Satscan purely spatial analysis). Map generated with QGIS 2.16 (QGIS Development Team, 2016. QGIS Geographic Information System. Open Source Geospatial Foundation Project. http://www.qgis.org/).

**Figure 5:**
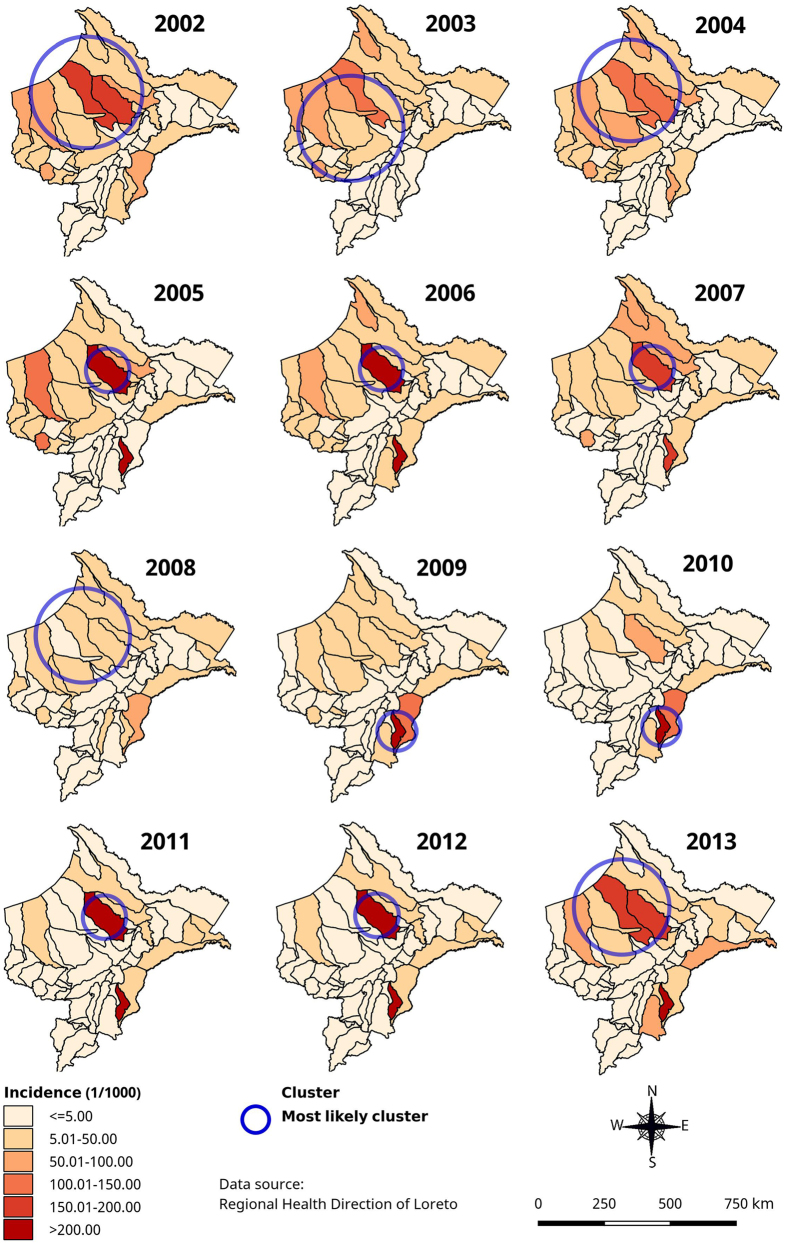
Annual *P. falciparum* incidence and adjusted most likely significant spatial clusters at district level in Loreto Region: 2002–2013 (using Satscan purely spatial analysis). Map generated with QGIS 2.16 (QGIS Development Team, 2016. QGIS Geographic Information System. Open Source Geospatial Foundation Project. http://www.qgis.org/).

Annual malaria risk maps based on AIRs by district showed a high heterogeneity in time and space for both species in Loreto (Figs 3, 4 and 5). During the study period, the overall AIR by district (Fig. 3) varied from 0 to 1,643/1,000 with consistently high incidence (>250/1,000 pop) reported in Soplin district (Southeast of Loreto), and extremely low incidence (0–5/1000) in the southern districts of Ucayali province. A purely spatial analysis using overall annual malaria incidence rates and adjusting for population density, proportion of poor population and density of health facilities, identified a significant most likely cluster for each year. The latter was consistently situated in the north-northwest part of Loreto bordering with Ecuador (Adj. IRR ranging 2.96 from to 11.41), except in the period 2008–2010 (Adj. IRR ranging from 10.78 to 14.84) when it included the southeastern districts of Soplin, Tapiche, Alto Tapiche, and Yaquerana, bordering with Brazil (Fig. 3). The adjusted most likely overall malaria space-time cluster included 12 northwestern districts with a 3.5-fold higher malaria incidence between January 2002 and August 2007 than in any other space-time cluster during the study period (Table 3). Additionally, one important secondary cluster was identified in Soplin district alone where malaria incidence was more than 30-fold higher (Adj.IRR = 31.84) between April 2004 and March 2010 compared to the other districts. Another secondary cluster was identified including the 5 western districts of Balsapuerto, Yurimaguas, Teniente César López and Santa Cruz were malaria incidence was significantly higher (Adj.IRR = 2.45) between January 2002 and September 2005.

*P. vivax* adjusted most likely spatial clusters (Fig. 4) overlapped with the overall malaria spatial clusters (Fig. 3) throughout the study period, except in 2012 when the cluster was located in the northeast part of Loreto including 15 districts around the border with Colombia. Similarly, the adjusted most likely *P. vivax* space-time cluster overlapped almost perfectly with the one for all malaria cases, and so did two of the adjusted secondary *P. vivax* clusters, *i.e.* the one in Soplin (Adj. IRR = 33.57) and the one including the 5 above mentioned western districts (Adj. IRR = 1.96) (Table 3). One additional *P. vivax* space-time cluster was identified from April 2012 to August 2013 including four districts of Ramon Castilla province were malaria incidence was almost 3-fold higher (Adj.IRR = 2.80) than in any other district in Loreto.

Regarding the *P. falciparum* most likely spatial clusters, though their size and the actual districts varied substantially between years, they followed the same locations than those of *P. vivax, i.e.* consistently located in the north-northwestern part of the region except in 2009–2010 when they were identified in the southeast districts of Soplin, Tapiche and Yaquerana (Fig. 5). Likewise, the *P. falciparum* space-time analysis also identified the most likely cluster from January 2002 to August 2007, but it was larger (22 districts) though largely overlapping the most likely *P. vivax* space-time cluster and extending more towards western part of Loreto (Adj. IRR = 4.05). Interestingly the secondary space-time cluster was also located in Soplin district from January 2005 to December 2010 where the *P. falciparum* incidence was almost 30-fold higher (Ajd.IRR = 28.8) (Table 3).

## Discussion

This 12-year retrospective analysis of the routinely collected malaria cases in Loreto Department allowed an in-depth characterization of significant space-time clusters of both *P. vivax* and *P. falciparum* malaria incidence in relation to applied control interventions. During the PAMAFRO project implemented between 2005 and 2010, the overall annual malaria incidence dropped by 80%, reaching pre-elimination level in most districts. However, after the end of the programme, both *P. vivax* and *P. falciparum* incidence increased dramatically, with more than a doubling of cases every year, returning to nearly the 2002 figures within only 3 years.

The Poisson regression analysis confirmed significant differences in malaria incidence between the three periods corresponding to the implementation of different malaria control activities. Indeed, during the first period (2002–2005), the overall malaria incidence first decreased, mainly due to a decrease in *P. falciparum* incidence. During this period, control efforts included the implementation of the new malaria treatment policies (*i.e.,* ACT for *P. falciparum* and the 7-day short-course primaquine regimen for *P. vivax*)^16^ together with PCD and immediate responses to malaria outbreaks (focalized active case detection and indoor residual spraying). These interventions were mainly implemented around Iquitos city, where the pressure for public health interventions by the population and local medias was high^17^. Between 2005 and 2010, the overall annual incidence rate decreased by nearly 80% for both species and this corresponded to the implementation of the PAMAFRO project with intensified and systematic control activities in communities and districts with the highest malaria risk in Loreto^5^. The third period of rapid malaria resurgence (2011–2013) corresponded to the post-PAMAFRO period, when malaria control activities were shifted under the responsibility of the MoH. However, the latter does not currently have specific resources for malaria control, and most of the budget allocated to the “control of transmissible diseases” has been relocated to more pressing public health emergencies such as dengue, after assuming that malaria was “under control” following the successful results of the PAMAFRO project^18^.

It is noteworthy that at the end of PAMAFRO, from July 2010 to February 2011, monthly malaria incidences were consistently low or very close to 1/1,000 suggesting that Loreto was near to achieve malaria elimination had the interventions been continued. The rapidity at which malaria returned to pre-project levels is striking, though this has been previously reported to a lesser extent in other settings^19^,^20^,^21^. This emphasizes the need to sustain elimination efforts once they have been started. The rapid upsurge observed since 2011 could be partly explained by the large proportion of asymptomatic and sub-microscopic malaria infections in the Peruvian endemic areas^22^,^23^,^24^,^25^. These infections, undetected by routine PCD, are likely to sustain and even increase malaria transmission when ecological conditions are favourable and/or control efforts are relaxed^22^,^26^. This was probably the case in 2011, when reported malaria incidence reached extremely low levels, and the pressure of intensified control measures was suddenly removed, possibly leaving the hidden parasite reservoir free to rapidly expand. The situation was further worsened by the unusually heavy rains occurring in 2011 and 2012^18^.

The northern, eastern and south-eastern districts of Loreto, mainly at international borders, were those reporting the highest malaria incidence throughout the study period. More specifically, the most likely space-time cluster was identified between 2002 and 2007, both for *P. vivax* and *P. falciparum* and with adjusted IRRs of 3.46 and 4.05, respectively While it included 22 districts for *P. falciparum,* covering most of the northwest and central part of Loreto, it also overlapped almost completely (10/12 districts) with the *P. vivax* space-time cluster and mainly the districts bordering with Ecuador. As previously mentioned, the period 2002–2007 corresponded to the episodically MoH-implemented control measures mainly in districts around Iquitos, and to the first two years of PAMAFRO project implementation whose impact on malaria transmission were more substantial in the following years.

**Figure 6:**
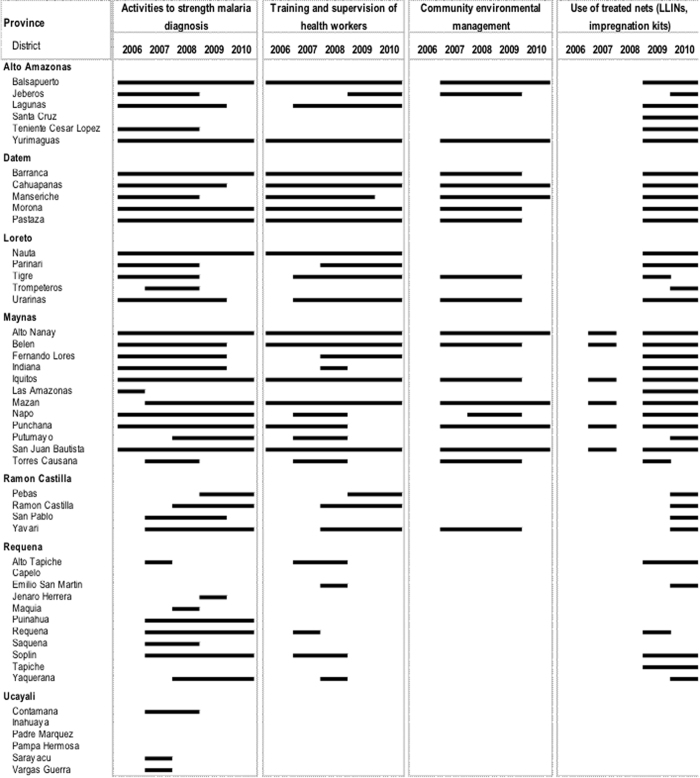
Main malaria control interventions by district and year implemented during the PAMAFRO project in Loreto Region.

The south-eastern district of Soplin was a consistent significant secondary cluster of both *P. vivax* (2004–2009) and *P. falciparum* (2005–2010) malaria cases with Adj.IRRs as high as 33.6 and 28.8, respectively. Interestingly, Soplin district was among the districts where PAMAFRO control interventions started later and lasted for shorter times in comparison with districts located in Alto Amazonas, Datem, Loreto and Maynas provinces (Fig. 6). Moreover, as shown by the adjusted Poisson regression analysis, there was a negative association between malaria incidence rates and the number of malaria interventions implemented in Loreto districts.

Between April 2012 and August 2013, a significant secondary space-time cluster of *P. vivax,* malaria cases was identified in the eastern province of Ramon Castilla. Beside the fact these districts were also among those where PAMAFRO control interventions started the latest and for the shortest duration, the recent proliferation of illegal economical activities such as logging and cultivation of coca plants may also partly explain the increasing malaria incidence in the last two years of the study period. Indeed, according to the Peruvian Government and United Nations Office on Drugs and Crime (UNODC), Loreto is the third region in Peru producing coca leafs for drug trafficking, and Ramon Castilla province, situated at the border with Brazil and Colombia, includes the districts with most most illegal cultivation lands^27^. This type of new economic activities increases the risk of human-mosquitoes contacts, but also generates important population movements to the Eastern border areas, including non-immune populations from non-malaria endemic regions of Peru, which are more likely to develop clinical malaria. Moreover, the illegal character of their activities may also negatively influence the health seeking behaviour of these migrant workers as previously described in malaria endemic areas of Colombia and Cambodia^28^,^29^.

Despite some variations across districts, the purely temporal analysis confirmed the occurrence of a single 5–6 month higher transmission period in the region, usually from February to July. This period corresponds to the second half of the rainy season and the beginning of the dry season, when the interactions between the different ecological and human factors are optimal for malaria transmission. Indeed *An. darlingi* is breeding in stagnant waters with floating vegetation along river margins in forested areas, especially in deforested and disturbed places close to forest areas where people live and work^8^,^9^. Entomological studies have shown that the vector density starts increasing in February, reaches maximum levels in April, and then declines progressively until July^7^. During this period, people with permanent outdoor economic activities (such as subsistence agriculture and fishing), and seasonal forest economic activities starting mainly at the beginning of the dry season (such as coaling and lumbering) are at increased risk of malaria infection^10^. Regarding species-specific temporal scan statistic, the *P. falciparum* malaria risk period started and finished slightly sooner than the one for *P. vivax,* as this has also reported in other countries were both species are endemic^30^. The observed variations in the start and duration of the transmission season across districts might be critical for planning future interventions aiming at reducing the parasite reservoir during the dry season such as focal screening and treatment (FSAT)^31^. Indeed, one of the main challenges currently faced by the MoH is the capacity to integrate contextualised ecological and human parameters in order to maximise the efficiency of future control and elimination interventions.

The main limitations of our study are inherent to the method used for the space-time analysis, especially the scale (district) at which data were available with only a single malaria incidence time point per district. Indeed, a breakdown of the monthly malaria incidence rates by community would have resulted in a more precise space-time analysis, but those rates were not available at community level. However, this limitation was partially addressed by the systematic adjustment for population density and therefore we believe our results are valid. Despite being recognized as a powerful statistical tool frequently used to analyse disease spatial and spatio-temporal patterns^32^, it is difficult to determine an optimal set of scaling parameters for the SaTScan analysis, especially for the maximum window size. Indeed, large maximum window sizes (as 50% of the total population used in our study) can hide small, homogeneous clusters within the larger and more heterogeneous ones, while on the other hand, small maximum windows sizes can miss significant regional-level clusters^13^. Other limitations were related to the use of retrospective data and the non-inclusion of other determinants of malaria transmission in the analysis (*e.g* age, location, socio-economic status, vegetation coverage, etc.) which were not available. Further research is needed to identify local factors of transmission within the clusters (at community level) in order to provide refined information to improve malaria control and elimination activities at district and provincial level and to better target the allocation of resources.

## Materials and Methods

### Study area and population

The Loreto Region (Fig. 1) is located in the northeast of Peru where it borders Ecuador, Colombia and Brazil. Covering almost one-third of the Peruvian territory (28.7%), the region is divided into 51 districts grouped into 7 provinces. Only 49 districts were included in the analysis, since Andoas and Teniente Clavero Lopez districts were only created after 2004. The region is called “Amazonic plain” (61 to 220 m elevation above sea level), and is covered by tropical rainforest and an extensive fluvial network connecting to the Amazon River^33^. The vast territory of the border region includes 11 districts, six of which are bordering with Ecuador (Morona, Pastaza, Tigre, Trompeteros, Napo and Torres Causana), two with Colombia (Putumayo and Ramón Castilla) and three with Brazil (Yavarí, Yaquerana, and Alto Tapiche). Iquitos city, the capital of Loreto, is the most important urban area within the region and is accessible only by boat or by plane. According to the 2007 census, the Loreto population totalled 891,732 inhabitants with more than one third (38.6%) below the age of 15 years, and about one third (34.6%) living in rural areas^34^. The local economy varies according to the different areas with indigenous and rural towns mainly depending on natural resources exploitation such as agriculture and fishing^35^, and bigger towns focusing on commercial activities^36^. The climate is warm and humid with traditionally the rainy season lasting from November to May and the dry season from June to October^33^. Precipitations are present throughout the year (annual average of 2,500 mm) with a usual peak in March (360 mm) and a minimum in July (50–100 mm), though there have been substantial variations between districts and in the recent years^37^. *An. darlingi*, an anthropophilic and exo/endophilic species, is considered as the main malaria vector in the region^6^,^7^,^38^.

### Malaria control activities in Loreto (2002–2013)

Following drug efficacy studies in the late 1990 s and early 2000 s confirming *P. falciparum* resistance to CQ or SP, the MoH decided to introduce in 2001 the ACT based on mefloquine (MQ)-artesunate (AS) as the first line treatment for *P. falciparum* in the Amazon Region^16^. Radical treatment guidelines for *P. vivax* malaria were also adjusted to a 7-day regimen with increased daily dose (0.5- instead of 0.25 mg/kg/day) of primaquine (PQ) following adherence problems to the usually 14-day regimen recommended by WHO^39^. Although the implementation of ACT in the region was initially (2002–2004) limited due to poor accessibility to many malaria endemic communities, it improved from 2005 onwards following the implementation of the trans-border “PAMAFRO” project^5^. During the 2002–2005 period, malaria control activities were essentially based on passive case detection (PCD), and response to malaria outbreaks with focalized active detection of symptomatic cases (ACD) and indoor residual spraying (IRS). Between October 2005 and September 2010, PAMAFRO was simultaneously implemented at the border areas of Venezuela, Colombia, Ecuador, and Peru. Common control strategies in those areas^5^, included: *a)* the strengthening of malaria diagnosis (*i.e.,* purchase of new microscopes, maintenance of existing microscopes, regular training and evaluation of microscopists, introduction of rapid diagnostic tests (RDTs) in remote areas, etc.)^40^*; b)* the improvement of malaria case-management (*i.e.,* purchase and distribution of antimalarial drugs, training of health workers, supervision of prescribing practices, monitoring treatment compliance, etc.); *c)* the encouragement of community participation in environmental management (*i.e.,* health education and promotion campaigns, technical and financial support of community environmental management activities, etc.) and *d)* the use of insecticide treated nets (*i.e.,* distribution of long lasting insecticide nets (LLINs), use of re-treatment insecticide kits)^41^.

During the PAMAFRO project the implementation of control interventions was not uniform in time and intensity across districts (Fig. 6). After the end of PAMAFRO project, the MoH was unable to maintain the same level of activities due to limited human and financial resources^18^. Therefore, malaria control activities are currently limited to PCD at health facilities and ACD in communities with high incidence of malaria cases (“hotspots”).

### Data collection

Retrospective data on annual malaria incidence by species were obtained from the Peruvian Regional Ministry of Health, Epidemiological Bureau - Dirección Regional de Salud Loreto (DIRESA Loreto). In Peru, malaria is a mandatory notifiable disease and is reported weekly to the health information system (HIS). At community level, patients presenting at health facilities with suspected malaria are systematically screened by microscopy (thick and thin smears) and, if positive, treated following national guidelines. Malaria cases identified at health facilities are reported to the district, provincial and regional level. At each level, data from the different sources (health facilities and hospitals) are consolidated and updated weekly in electronic databases. In this study, malaria cases were aggregated by month from January 2002 to December 2013 for each of the 49 study districts. Projected district population sizes at June 30^th^ of each year were obtained from the National Institute of Statistics and Informatics. Those projections took into account the estimated resident population in two national censuses conducted in 1993 and 2007^42^.

Monthly climate time series were obtained from the Peruvian National Service of Meteorology and Hydrology, and included mean temperature (in Celsius degrees, °C), cumulated rainfall (in millimetres, mm) and mean relative humidity (in percentage, %) collected from 13 meteorological stations covering Loreto Region between 2002 and 2013.

### Data analysis

#### Mapping and analysis of annual malaria incidences

Data were entered and cleaned in Excel 2010 (Microsoft Corp, USA). Annual incidence rates (AIRs, per 1000 population) were calculated for each of the 49 districts and over the twelve-year period (2002–2013). Subsequently, districts were grouped into the following six categories: *i)* 0–5.00 cases/1000 pop/y; *ii)* 5.01–50.00/1000 pop/y; *iii)* 50.01–100.00/1000 pop/y; *iv)* 100.01–150.00/1000 pop/y; *v)* 150.01–200.00/1000 pop/y; *vi)* >200.00/1000 pop/y. Maps showing AIRs, per district were generated using QGIS^TM^ v.2.16 (QGIS developer team, Open Source Geospatial Foundation). Two separated Poisson regression analyses were performed considering the number of yearly malaria cases per district (in total and by species) as a dependent variable, and the yearly total population per district (logarithmic scale) as the population at risk. The first analysis included the year of the reported malaria cases as independent variable to identify significant changes in malaria incidence over time, while the second analysis included the number of malaria control interventions implemented during the PAMAFRO period per district. Interventions were grouped per district in a specific year, and then stratified as follows: *i)* no intervention supported by PAMAFRO; *ii)* only one intervention (either the strengthening of malaria diagnosis and case management, or the implementation of LLINs); and *iii)* two interventions simultaneously. The Poisson analysis explored the potential effect of those interventions on the annual malaria incidence at district level without and with adjustment for the following yearly variables for each district: *i)* climatic variables (*i.e.* mean temperature in °C, cumulated rainfall in mm, mean humidity in %) estimated through an inverse distance weighting (IDW) spatial interpolation^43^ using measurements of Peruvian National Service of Meteorology and Hydrology from 13 stations distributed over Loreto territory; *ii*) the population density calculated by dividing the population by the total district area (km^2^); *iii)* the population living in rural areas, and *iv)* the population living in poverty, both obtained from the National Institute of Statistics and Informatics^44^; and *v)* the number of health facilities in the district obtained from DIRESA Loreto. AIRs, incidence rate ratios (IRRs) (in comparison with the year 2002 or with no malaria control interventions supported by PAMAFRO), and 95% confidence intervals were calculated using R v3.1.3 software (R Development Core Team, R Foundation for Statistical Computing, Austria).

#### Cluster analysis

Scan statistics, both purely temporal and spatial as well as space-time, were applied to identify malaria incidence clusters at district level over the 12-year study period using SaTScan^TM^ software v.9.4.2 (M Kulldorf and Information Management Services Inc, USA). The purely temporal cluster analysis was performed under the null hypothesis that malaria incidence was equally distributed over the entire study period. The software established different windows (intervals) moving in one (time) dimension. Each window represented a possible cluster for which an IRR (malaria incidence inside/outside window) was calculated^45^. A Poisson probability model with a cut-off p-value < 0.05 was used, time aggregation was set to 1 month and the maximum temporal cluster size was set to the default value ≤ 50% of the study period^46^.

The purely spatial and space-time analyses were performed without and with adjustment for the following covariates at district level, previously classified into quartiles: *i)* population density, *ii)* proportion of poor population as defined elsewhere^44^, and *iii)* density of health services (i.e., number of health facilities/10000 inhabitants). The purely spatial analysis was performed under the null hypothesis that all districts in Loreto Region have equal risk of malaria incidence. Different windows with varying size, from zero to a maximum radius of less than 50% of the total population, were allowed to move across the study area. Each circle was a candidate cluster for which the likelihood ratio (LLR) and the IRR were obtained. The circular window with the highest LLR was defined as the most likely cluster if *p*-value < 0.05^46^,^47^.

The space-time analysis was performed under the null hypothesis that the incidence of malaria was the same in all districts and over time, with cylindrical windows having a circular geographic base and height corresponding to the time scale in months. The radius of each circular base was allowed to vary in size, to include up to as many as 50% of the total population. Comparably, the height of the cylinder varied in size up to a maximum of 50% of the study period with a time precision of one month. An unlimited number of overlapping cylinders with different dimensions were obtained jointly covering the Loreto Region, each cylinder corresponding to a possible space-time cluster. The statistical significance of the clusters were tested through Monte Carlo simulations, establishing 999 replications (the default value of the software) to achieve suitable power, the null hypothesis being rejected if the resulting p-value was below 0.05. For each cluster (purely spatial or space-time), the LLR was calculated and the most likely cluster defined as the cylinder with the maximum LLR. Secondary clusters that did not overlap with the most significant cluster were also identified and ranked according to their LLR test statistic^48^.

## Additional Information

**How to cite this article**: Soto-Calle, V. *et al*. Spatio-temporal analysis of malaria incidence in the Peruvian Amazon Region between 2002 and 2013. *Sci. Rep.* **7**, 40350; doi: 10.1038/srep40350 (2017).

**Publisher's note:** Springer Nature remains neutral with regard to jurisdictional claims in published maps and institutional affiliations.

## Supplementary Material

Supplementary Information

## Figures and Tables

**Table 1:** Evolution of the overall and species-specific annual incidence rates (AIR).

Year	Total				*P. vivax*				*P. falciparum*			
	AIR	IRR	(CI 95%)		AIR	IRR	(CI 95%)		AIR	IRR	(CI 95%)	
2002	58.09	1			40.77	1			17.32	1		
2003	51.96	0.89	0.55	1.45	40.44	0.99	0.61	1.62	11.52	0.67	0.35	1.25
2004	47.26	0.81	0.49	1.34	36.30	0.89	0.54	1.47	10.96	0.63	0.32	1.24
2005	59.18	1.02	0.63	1.65	44.89	1.10	0.66	1.83	14.29	0.83	0.46	1.47
2006	48.86	0.84	0.52	1.36	40.55	0.99	0.61	1.61	8.32	0.48	0.27	0.87
2007	39.13	0.67	0.40	1.13	31.08	0.76	0.46	1.26	8.05	0.46	0.23	0.93
2008	25.77	0.44	0.26	0.75	21.12	0.52	0.31	0.87	4.65	0.27	0.14	0.53
2009	26.69	0.46	0.27	0.79	22.67	0.56	0.32	0.96	4.02	0.23	0.12	0.46
2010	11.59	0.20	0.11	0.35	9.33	0.23	0.13	0.41	2.26	0.13	0.07	0.25
2011	11.79	0.20	0.12	0.36	9.31	0.23	0.13	0.40	2.48	0.14	0.07	0.30
2012	25.02	0.43	0.24	0.76	21.08	0.52	0.29	0.92	3.94	0.23	0.11	0.45
2013	42.69	0.73	0.43	1.24	34.94	0.86	0.51	1.44	7.75	0.45	0.23	0.88

AIR = Annual incidence rates (/1000 population), IRR = incidence rate ratio compared to 2002 estimated from Poisson analysis.

**Table 2:** Temporal clusters of malaria cases in Loreto Region, 2002–2013.

	Total		*P. vivax*		*P. falciparum*	
	Cluster.period	IRR*	Cluster period	IRR*	Cluster period	IRR*
2002	Feb–Jul	1.47	Feb–Jul	1.46	Jan–Jun	1.59
2003	Feb–Jul	1.62	Feb–Jul	1.64	Feb–Jun	1.56
2004	May–Sep	1.36	May–Sep	1.40	Feb–Jul	1.24
2005	Feb–Jun	1.52	Feb–Jun	1.63	Feb–Jul	1.27
2006	Apr–Jun	1.91	Apr–Jun	1.95	Apr–Jun	1.70
2007	Mar–Aug	1.84	Mar–Aug	1.88	Mar–Aug	1.72
2008	Feb–Jul	1.50	Feb–Jul	1.61	Apr–May	1.20
2009	Mar–Aug	1.77	Jun–Aug	1.88	Mar–Jun	1.53
2010	Jan–Jun	1.62	Jan–Jun	1.71	Feb–Jul	1.45
2011	May–Sep	1.77	May–Sep	1.61	Jun–Sep	1.75
2012	May–Jul	2.18	May–Jul	2.33	Jul	1.89
2013	Apr–Jul	1.72	Apr–Jul	1.77	May–Jul	1.63

IRR = incidence rate ratio (compared to all other months) estimated by purely temporal analysis using Satscan. *All p-values were <0.001

**Table 3:** Significant malaria space-time clusters at district level in Loreto Region (2002–2013) using Satscan time space analysis.

Cluster	Districts	Time frame	N (%)	O (E)	IRR*
**Total**					
A	Tigre, Trompeteros, Alto Nanay, Napo, Urarinas, Mazan, Pastaza, Torres Causana, Nauta, Punchana, Lagunas, San Juan	Jan 2002–Aug 2007	318,752 (33.5%)	157,846 (61,439)	3.52
B	Soplin	Apr 2004–Mar 2010	653 (0.1%)	4,349 (138)	31.84
B*	Yurimaguas, Teniente Cesar Lopez, Santa Cruz, Balsapuerto	Jan 2002–Sep 2005	92,849 (9.8%)	26,256 (11,138)	2.45
***P. vivax***					
A	Tigre, Trompeteros, Alto Nanay, Napo, Urarinas, Mazan, Pastaza, Torres Causana, Nauta, Punchana, Lagunas, San Juan	Apr 2002–Aug 2007	318,752 (33.5%)	119,447 (46,490)	3.46
B	Soplin	Jan 2004–Dec 2009	653 (0.1%)	3,480 (105)	33.57
B	Ramon Castilla, San Pablo, Pebas, Yavari	Apr 2012–Aug 2013	58,959 (6.2%)	12,480 (58,959)	2.80
B	Yurimaguas, Teniente Cesar Lopez, Santa Cruz, Balsapuerto	Jan 2002–Aug 2005	92,849 (9.8%)	16,693 (92,849)	1.96
***P. falciparum***					
A	Pastaza, Morona, Trompeteros, Lagunas, Manseriche, Urarinas, Barranca, Cahuapanas, Tigre, Jeberos, Balsapuerto, Santa Cruz, Yurimaguas, Parinari, Alto Nanay, Teniente Cesar Lopez, Purinahua, Nauta, Napo, Torres Causana, Capelo, San Juan	Jan 2002–Aug 2007	375,039 (39.5%)	45,004 (17,993)	4.05
B	Soplin	Jan 2005–Dec 2010	653 (0.1%)	947 (653)	28.82

A: Most likely cluster; B: Secondary cluster; N: total population in the cluster; O: Number of observed cases in the cluster; E: number of expected cases in the cluster; IRR: Incidence rate ratio; *All p-values < 0.001.

## References

[b1] Dirección General de Epidemiología - Ministerio de Salud Perú. *Sala de Situación de Salud Perú: Semana epidemiológica No 01–2015*. - (MINSA, 2015).

[b2] Griffing S. M., Gamboa D. & Udhayakumar V. The history of 20th century malaria control in Peru. Malar. J. 12, 303 (2013). 24001096 10.1186/1475-2875-12-303 PMC3766208

[b3] Aramburu G. J., Ramal A. C. & Witzig R. Malaria re-emergence in the Peruvian Amazon region. Emerg. Infect. Dis. 5, 209–215 (1999). 10221872 10.3201/eid0502.990204 PMC2640690

[b4] Neyra D., Cabezas C., Trenton K. & Ruebush I. I. El Proceso de Adecuación y Cambio en la Política del Tratamiento de la Malaria por *Plasmodium falciparum* en el Perú, 1990–2001. Rev. Peru Med. Exp. Salud Publica 20 **(3),** 162–171 (2012).

[b5] Organismo Andino de Salud. PAMAFRO: *Compartiendo lecciones aprendidas*. (Organismo Andino de Salud, http://www.orasconhu.org/pamafro/compartiendo-lecciones-aprendidas-del-pamafro 2009).

[b6] Maheu-Giroux M. . Risk of malaria transmission from fish ponds in the Peruvian Amazon. Acta Trop. 115, 112–118 (2010). 20188688 10.1016/j.actatropica.2010.02.011

[b7] Reinbold-Wasson D. D. . Determinants of Anopheles seasonal distribution patterns across a forest to peri-urban gradient near Iquitos, Peru. Am. J. Trop. Med. Hyg. 86, 459–463 (2012). 22403317 10.4269/ajtmh.2012.11-0547 PMC3284362

[b8] Vittor A. Y. . The effect of deforestation on the human-biting rate of *Anopheles darlingi*, the primary vector of Falciparum malaria in the Peruvian Amazon. Am J Trop Med Hyg 74, 3–11 (2006). 16407338

[b9] Vittor A. Y. . Linking deforestation to malaria in the Amazon: characterization of the breeding habitat of the principal malaria vector, *Anopheles darlingi*. Am J TropMed Hyg 81, 5–12 (2009). PMC3757555 19556558

[b10] Chuquiyauri R. . Socio-demographics and the development of malaria elimination strategies in the low transmission setting. Acta Trop. 121 (2012). 10.1016/j.actatropica.2011.11.003 PMC3294046 22100446

[b11] Peeters Grietens K. . Traditional nets interfere with the uptake of long-lasting insecticidal nets in the Peruvian Amazon: the relevance of net preference for achieving high coverage and use. PloS One 8, e50294 (2013). 23300943 10.1371/journal.pone.0050294 PMC3534704

[b12] Peeters Grietens K. . Adherence to 7-day Primaquine Treatment for the Radical Cure of *P. vivax* in the Amazon Region. Am. J. Trop. Med. Hyg. 82, 1017–1023 (2015). 10.4269/ajtmh.2010.09-0521 PMC2877405 20519594

[b13] Aamodt G., Samuelsen S. O. & Skrondal A. A simulation study of three methods for detecting disease clusters. Int. J. Health Geogr. 5, 1–11 (2006). 16608532 10.1186/1476-072X-5-15 PMC1468395

[b14] Robertson C. & Nelson T. A. Review of software for space-time disease surveillance. Int. J. Health Geogr. 9, 1–8 (2015). 10.1186/1476-072X-9-16 PMC2848213 20226054

[b15] Song C. & Kulldorff M. Power evaluation of disease clustering tests. Int. J. Health Geogr. 2, 1–8 (2015). 10.1186/1476-072X-2-9 PMC333429 14687424

[b16] Williams H. A., Vincent-Mark A., Herrera Y. & Chang O. J. A retrospective analysis of the change in anti-malarial treatment policy: Peru. Malar. J. 8, 85 (2009). 19400953 10.1186/1475-2875-8-85 PMC2684118

[b17] Consultations - GMAP 2. Available at: http://www.gmap2.org/english/consultations/. (Accessed: 21st December 2015).

[b18] Rosas-Aguirre A. Understanding malaria transmission dynamics for malaria control and elimination in Peru. PhD Thesis. (Université Catholique de Louvain, 2015).

[b19] Cohen J. M. . Malaria resurgence: a systematic review and assessment of its causes. Malar. J. 11, 122 (2012). 22531245 10.1186/1475-2875-11-122 PMC3458906

[b20] Oliveira-Ferreira J. . Malaria in Brazil: an overview. Malar. J. 9, 115 (2010). 20433744 10.1186/1475-2875-9-115 PMC2891813

[b21] Xu B.-L., Su Y.-P., Shang L.-Y. & Zhang H.-W. Malaria control in Henan Province, People’s Republic of China. Am. J. Trop. Med. Hyg. 74, 564–567 (2006). 16606984

[b22] da Silva-Nunes M. . Amazonian malaria: Asymptomatic human reservoirs, diagnostic challenges, environmentally-driven changes in mosquito vector populations, and the mandate for sustainable control strategies. Acta Trop. 121, 281–291 (2012). 22015425 10.1016/j.actatropica.2011.10.001 PMC3308722

[b23] Vinetz J. M. & Gilman R. H. Asymptomatic Plasmodium parasitemia and the ecology of malaria transmission. Am. J. Trop. Med. Hyg. 66, 639–640 (2002). 12224566 10.4269/ajtmh.2002.66.639

[b24] Rosas-Aguirre A. . Hotspots of Malaria Transmission in the Peruvian Amazon: Rapid Assessment through a Parasitological and Serological Survey. PLoS ONE 10, e0137458 (2015). 26356311 10.1371/journal.pone.0137458 PMC4565712

[b25] Rosas-Aguirre A. . Assessing malaria transmission in a low endemicity area of north-western Peru. Malar. J. 12, 339 (2013). 24053144 10.1186/1475-2875-12-339 PMC3849384

[b26] Sturrock H. J. W. . Targeting Asymptomatic Malaria Infections: Active Surveillance in Control and Elimination. PLoS Med. 10 (2013). 10.1371/journal.pmed.1001467 PMC3708701 23853551

[b27] Comision Nacional para el Desarrollo y Vida sin Drogas (DEVIDA), U. N. O. on D. and C. (UNODC). Peru: Monitoreo de Cultivos de Coca 2013. (DEVIDA, 2014).

[b28] Chaparro P., Padilla J., Vallejo A. F. & Herrera S. Characterization of a malaria outbreak in Colombia in 2010. Malar. J. 12, 330 (2013). 24044437 10.1186/1475-2875-12-330 PMC3848715

[b29] Peeters Grietens K. . Characterizing Types of Human Mobility to Inform Differential and Targeted Malaria Elimination Strategies in Northeast Cambodia. Sci. Rep. 5, 16837 (2015). 26593245 10.1038/srep16837 PMC4655368

[b30] Hui F. M. . Spatio-temporal distribution of malaria in Yunnan Province, China. Am. J. Trop. Med. Hyg. 81, 503–509 (2009). 19706922

[b31] Rosas-Aguirre A. . Modelling the potential of focal screening and treatment as elimination strategy for Plasmodium falciparum malaria in the Peruvian Amazon Region. Parasit. Vectors 8, 261 (2015). 25948081 10.1186/s13071-015-0868-4 PMC4429469

[b32] Bousema T. & Baidjoe A. in Ecology of parasite-vector interactions 197–220 (Wageningen Academic Publishers, 2013).

[b33] Instituto de Investigaciones de la Amazonia Peruana (IIAP) - Proyecto Diversidad Biológica de la Amazonía Peruana (BIODAMAZ). Diversidad de la Vegetación de la Amazonía Peruana Expresada en un Mosaico de Imágenes de Satélite. **Documento Técnico N**^**o**^**12,** (IIAP, 2011).

[b34] Instituto Nacional de Estadística e Informática (INEI). Perú: Crecimiento y distribución de la población 2007. Censo Nacional 2007: XI de Población y VI de Vivienda. - (INEI, 2012).

[b35] Ministerio de Salud - Dirección General de Epidemiología. Análisis de la Situación de Salud del Perú, ASIS 2010. (MINSA, 2012).

[b36] Instituto Nacional de Estadística e Informática - INEI. *Boletín Departamental No 16: Loreto* (2013).

[b37] Dirección Regional de Transportes y Comunicaciones- Gobierno Regional de Loreto. Plan Vial Departamental Participativo de Loreto. (Gobierno Regional de Loreto, 2012).

[b38] Turell M. J. . Seasonal distribution, biology, and human attraction patterns of mosquitoes (Diptera: Culicidae) in a rural village and adjacent forested site near Iquitos, Peru. J. Med. Entomol. 45, 1165–1172 (2008). 19058644 10.1603/0022-2585(2008)45[1165:sdbaha]2.0.co;2

[b39] Williams H. . A retrospective analysis of the change in anti-malarial treatment policy. Peru. Malar. J. 8, 85 (2009). 19400953 10.1186/1475-2875-8-85 PMC2684118

[b40] Rosas-Aguirre Á. . [Use of standardized blood smear slide sets for competency assessment in the malaria microscopic diagnosis in the Peruvian Amazon]. Rev. Peru. Med. Exp. Salud Pública 27, 540–547 (2010). 21308193 10.1590/s1726-46342010000400008

[b41] Rosas-Aguirre A. . [Long-lasting insecticide - treated bednet ownership, retention and usage one year after their distribution in Loreto, Peru]. Rev. Peru. Med. Exp. Salud Pública 28, 228–236 (2011). 21845302 10.1590/s1726-46342011000200009

[b42] Instituto Nacional de Estadística e Informática (INEI). Perú: Estimaciones y Proyecciones de la Población por Sexo según Departamento, Provincia y Distrito, 2000–2015. Boletin No 18. - (INEI, 2011).

[b43] Shepard D. A two-dimensional interpolation function for irregularly-spaced data. Proc. 1968 A. C. M. Nat. Confi. 517–524 (1968).

[b44] Instituto Nacional de Estadística e Informática (INEI). Mapa de Pobreza Provincial y Distrital 2013.- (INEI, 2015).

[b45] Kulldorff M. SaTScan^TM^ UserGuide for version 9.4.2. (2010).

[b46] Kulldorff M. A spatial scan statistic. Commun. Stat. Theory. Methods. 26(6), 1481–1496 (2012).

[b47] Kulldorff M. & Nagarwalla N. Spatial disease clusters: detection and inference. Stat. Med. 14, 799–810 (2015). 10.1002/sim.4780140809 7644860

[b48] Kulldorff M., Athas W. F., Feurer E. J., Miller B. A. & Key C. R. Evaluating cluster alarms: a space-time scan statistic and brain cancer in Los Alamos, New Mexico. Am. J. Public Health 88, 1377–1380 (1998). 9736881 10.2105/ajph.88.9.1377 PMC1509064

