# Supplementary material for: Spatio-temporal analysis of malaria incidence in the Peruvian Amazon Region between 2002 and 2013
Source: Sci Rep. 2017 Jan 16;7:40350. doi: 10.1038/srep40350 (PMC5238441; doi:10.1038/srep40350)
Supplement: Supplementary Information [file srep40350-s1.pdf]

Supplementary information for

Spatio-temporal analysis of malaria incidence in the Peruvian Amazon Region between 2002 and 2013

Veronica Soto-Calle[+], Angel Rosas-Aguirre[+], Alejandro Llanos-Cuentas , Emmanuel Abatih, Redgi DeDeken, Hugo Rodriguez, Anna Rosanas- Urgell, Dionicia Gamboa, Umberto D´Alessandro, Annette Erhart[+], Niko Speybroeck[+]

+These authors contributed equally to this work

This document includes: -Supplementary Table S1

Supplementary Table S1. Temporal clusters of malaria cases at district level (Pastaza, Balsapuerto, Tigre, Alto Nanay, Yavari, Soplin, Sarayacu) in Loreto Region, 2002-2013

	**All species**			***Plasmodium vivax***			***Plasmodium falciparum***		
	**Cluster**	RR	p	**Cluster**	**RR**	p	**Cluster**	**RR**	p
**Pastaza District**									
**2002**	Jan	2.10	0.001	Nov-Dec	1.66	0.001	Jan	2.81	0.001
**2003**	Aug-Oct	1.96	0.001	Aug-Oct	1.55	0.001	Jul-Nov	2.53	0.001
**2004**	Feb-Apr	1.84	0.001	Feb-Apr	2.39	0.001	Feb-Jun	1.48	0.001
**2005**	Jun	1.54	0.001	Nov-Dec	2.53	0.001	Apr-Sep	1.68	0.001
**2006**	Apr-Aug	1.94	0.001	Apr-Aug	1.76	0.001	Apr-Sep	2.69	0.001
**2007**	Jun-Sep	2.48	0.001	Jun-Sep	3.27	0.001	May-Sep	1.97	0.001
**2008**	Aug	1.36	0.001	Jan-Feb	1.30	0.001	Aug	1.83	0.001
**2009**	Jan-Jun	3.48	0.001	Jan-Jun	2.93	0.001	Jan-Jun	8.09	0.001
**2010**	Sep	2.13	0.001	Sep	2.32	0.001	May-Aug	2.27	0.231
**2011**	Apr-Jul	2.02	0.001	Apr-Jul	2.05	0.001	Dec	21.55	0.001
**2012**	Jul-Dic	1.87	0.001	Nov	2.04	0.001	Jul-Dec	4.17	0.001
**2013**	Mar-Apr	1.33	0.001	Sep-Nov	1.39	0.001	Jan-Jun	2.73	0.001
**Balsapuerto District**									
**2002**	Sep	1.45	0.001	Sep-Oct	1.45	0.001	Jan-Apr	1.42	0.001
**2003**	Jan-May	2.48	0.001	Jan-May	2.88	0.001	Feb-May	2.05	0.001
**2004**	Feb-May	1.58	0.001	Mar-May	2.14	0.001	Jan-Feb	1.60	0.001
**2005**	Jul-Sep	1.46	0.001	Jul-Sep	1.40	0.001	Apr-Sep	1.65	0.001
**2006**	Jul-Dec	1.42	0.001	Aug-Sep	1.36	0.001	Jul-Dec	1.99	0.001
**2007**	Jan-May	2.36	0.001	Jan-May	2.37	0.001	Jan-May	2.36	0.001
**2008**	Jul-Dec	1.96	0.001	Jul-Dec	1.92	0.001	Jul-Dec	2.02	0.001
**2009**	Jan-May	3.54	0.001	Jan-May	3.14	0.001	Jan-Apr	5.69	0.001
**2010**	Jan-Apr	4.44	0.001	Jan-Apr	5.34	0.001	Mar-May	3.96	0.455
**2011**	Oct	5.39	0.022	Oct	5.39	0.022	No cluster found		
**2012**	Sep-Dec	8.00	0.057	Sep-Dec	8.00	0.057	No cluster found		
**2013**	Feb-Mar	4.28	0.001	Feb-Mar	4.28	0.001	No cluster found		
**Tigre District**									
**2002**	Jun-Nov	2.71	0.001	Jul-Oct	3.34	0.001	Jun-Nov	2.46	0.001
**2003**	Sep-Nov	1.71	0.001	Jul-Nov	2.30	0.001	May	2.67	0.001
**2004**	Jul-Sep	2.15	0.001	Jul	1.96	0.001	Jul-Oct	4.35	0.001
**2005**	Jul-Sep	2.11	0.001	Jul-Sep	2.17	0.001	Aug-Nov	2.92	0.001
**2006**	May-Oct	2.06	0.001	May-Oct	2.08	0.001	Jul-Oct	2.48	0.001
**2007**	Mar-Apr	3.09	0.001	Mar-Apr	3.13	0.001	Mar-Apr	2.91	0.001
**2008**	Oct-Dec	2.34	0.001	Oct-Dec	2.37	0.001	Nov-Dec	2.41	0.001
**2009**	Apr-Jun	2.37	0.001	Mar-Jun	2.28	0.001	Apr-Sep	3.50	0.001
**2010**	Jan	2.10	0.183	Jan	2.31	0.092	No cluster found		
**2011**	Aug-Dec	11.61	0.001	Aug-Dec	12.31	0.001	Nov	10.63	0.001
**2012**	Mar-Jul	3.69	0.001	Mar-Jul	3.74	0.001	Mar-Jul	2.55	0.102
**2013**	Jun-Nov	3.99	0.001	Jun-Nov	3.82	0.001	Jun-Nov	4.30	0.001
**Alto Nanay District**									
**2002**	Mar-Jul	2.31	0.001	Mar-Aug	2.62	0.001	Mar-Jun	2.36	0.001
**2003**	Mar-Jul	1.61	0.001	May-Oct	1.46	0.005	Apr-Jul	2.72	0.001
**2004**	May	3.82	0.001	May	3.72	0.001	Apr-Jun	3.35	0.001
**2005**	Nov-Dic	2.28	0.001	Nov-Dec	2.17	0.001	Dec	3.16	0.001
**2006**	Jan-Jun	3.06	0.001	Jan-Jun	2.17	0.001	Jan-Jun	10.40	0.001
**2007**	Mar-Aug	3.57	0.001	Mar-Aug	4.00	0.001	May-Oct	3.15	0.001
**2008**	Nov	2.00	0.001	Nov	1.95	0.001	Nov	2.17	0.218
**2009**	Aug-Dic	4.79	0.001	Aug-Dec	5.06	0.001	Nov	4.55	0.005
**2010**	Feb	2.96	0.001	Dec	1.90	0.008	Feb	4.61	0.001
**2011**	Jan	2.29	0.001	Jan	1.62	0.005	Jan-Feb	2.96	0.001
**2012**	Jul-Dic	1.68	0.001	Jul-Dec	1.53	0.001	Nov-Dec	2.28	0.001
**2013**	Apr-Aug	1.35	0.001	Jun-Aug	1.63	0.001	Jan	1.81	0.002
**Yavari District**									
**2002**	Jul-Oct	13.71	0.001	Jul-Oct	13.69	0.001	Jul-Oct	13.77	0.001
**2003**	Apr-Aug	3.83	0.001	Apr-Au	3.28	0.001	Apr-Jul	7.65	0.001
**2004**	Feb-Jul	1.55	0.002	May-Sep	1.48	0.010	Mar-Aug	6.18	0.001
**2005**	Jun-Jul	5.44	0.001	Jun-Jul	4.41	0.001	Jun-Jul	12.46	0.001
**2006**	Jun-Jul	8.75	0.001	Jun-Jul	9.34	0.001	Jun	7.38	0.001
**2007**	Mar-Jul	3.70	0.001	Mar-Jul	4.03	0.001	Mar-May	3.83	0.001
**2008**	Jan-Apr	11.72	0.001	Jan-Apr	9.43	0.001	Jan-Apr	40.21	0.001
**2009**	Jul-Aug	4.49	0.001	Jul-Aug	5.11	0.001	Jun-Aug	3.34	0.001
**2010**	Jun	2.51	0.001	Jun-Jul	1.84	0.001	May-Jun	2.99	0.001
**2011**	Jun-Jul	2.51	0.001	Jun-Jul	2.35	0.001	Jun-Jul	3.45	0.001
**2012**	Mar-Aug	2.38	0.001	Mar-Aug	2.60	0.001	May-Sep	1.81	0.001
**2013**	Apr-Jul	3.01	0.001	Mar-Aug	3.08	0.001	Apr-Jul	3.75	0.001
**Soplin District**									
**2002**	Jun-Aug	3.00	0.001	Jun-Jul	3.15	0.001	Aug	10.77	0.008
**2003**	Feb-Apr	2.50	0.001	Feb-Apr	2.52	0.001	No cluster found		
**2004**	Nov	2.00	0.001	Nov	2.16	0.001	Mar-Jun	11.00	0.001
**2005**	Oct-Dec	2.67	0.001	Oct-Dec	2.46	0.001	Oct-Dec	3.32	0.001
**2006**	Feb-Jul	1.75	0.001	Feb-Jul	1.48	0.001	Jan-Apr	2.93	0.001
**2007**	Mar-Jun	2.24	0.001	Mar-Jun	1.93	0.001	Mar-Jun	5.12	0.001
**2008**	Dec	5.55	0.001	Dec	5.73	0.001	Nov	18.67	0.003
**2009**	Nov	2.00	0.001	Jan-Jun	2.66	0.001	Oct-Dec	126.86	0.001
**2010**	Feb-May	2.27	0.001	Feb-Apr	2.22	0.001	Jan-May	3.79	0.001
**2011**	Jan-Jun	1.57	0.006	Jan-Jun	1.92	0.001	Dec	2.07	0.211
**2012**	Jul-Dec	2.82	0.001	Jun-Nov	2.00	0.001	Aug-Dec	12.07	0.001
**2013**	Jun-Jul	2.97	0.001	May-Jul	2.26	0.001	Jun-Jul	4.98	0.001
**Sarayacu District**									
**2002**	Nov	7.44	0.018	Nov	9.57	0.006	No cluster found		
**2003**	Jun-Aug	11.87	0.001	Jun-Aug	14.84	0.001	May-Aug	7.87	0.388
**2004**	Jun-Jul	3.21	0.091	Jun-Jul	3.08	0.153	No cluster found		
**2005**	Jan-Jun	2.64	0.325	Mar-Jun	3.98	0.118	No cluster found		
**2006**	Feb	7.22	0.154	Feb	9.03	0.118	No cluster found		
**2007**	No cluster found			No cluster found			No cluster found		
**2008**	No cluster found			No cluster found			No cluster found		
**2009**	Dec	7.18	0.455	Aug-Sep	4.98	0.660	No cluster found		
**2010**	Mar	41.56	0.001	Mar	41.56	0.001	No cluster found		
**2011**	Jan-Feb	16.42	0.001	Jan-Feb	19.71	0.001	No cluster found		
**2012**	Jan-Feb	4.08	0.467	Jan-Jun	6.07	0.345	No cluster found		
**2013**	No cluster found			No cluster found			No cluster found		

RR = Relative Risk
